# The Effects of Low-Level Helium–Neon (He–Ne) Laser Irradiation on Lipids and Fatty Acids, and the Activity of Energetic Metabolism Enzymes and Proteome in the Blastula Stage and Underyearlings of the Atlantic Salmon *Salmo salar*: A Novel Approach in Salmonid Restoration Procedures in the North

**DOI:** 10.3390/biom12010133

**Published:** 2022-01-14

**Authors:** Svetlana A. Murzina, Viktor P. Voronin, Maria V. Churova, Tatiana R. Ruokolainen, Natalia S. Shulgina, Dmitriy S. Provotorov, Olga V. Tikhonova, Nina N. Nemova

**Affiliations:** 1 Institute of Biology of the Karelian Research Centre of the Russian Academy of Sciences (IB KarRC RAS), 11 Pushkinskaya Street, 185910 Petrozavodsk, Russia; voronen-viktor@mail.ru (V.P.V.); mchurova@yandex.ru (M.V.C.); truok@krc.karelia.ru (T.R.R.); shulgina28@yandex.ru (N.S.S.); provotorov.ds@mail.ru (D.S.P.); nemova@krc.karelia.ru (N.N.N.); 2 Institute of Biomedical Chemistry (IBMC), 10 Pogodinskaya Street, 119121 Moscow, Russia; ovt.facility@gmail.com

**Keywords:** lipids, fatty acids, energetic metabolism, proteins, ontogenesis, He–Ne laser, laser irradiation, aquaculture

## Abstract

The effect of He–Ne laser irradiation on fishery parameters as well as on biochemical state, including the lipids and fatty acids, the activity of energy metabolism enzymes and the proteome in the blastula stage and in underyearlings of wild Atlantic salmon after irradiation at the cleavage stage/early blastula (considered as the stages when the cell has a high potential for differentiation) was studied. Low mortality rates of eggs were determined during embryogenesis, as well as increased weight gain and lower morality rates of underyearlings in the experimental group. This is confirmed by changes in a number of interrelated indicators of lipid metabolism: a decrease in total lipids content, including diacylglycerols, triacylglycerols, cholesterol esters, and the phospholipids content remained unchanged. The embryos in the blastula stage (experimental group) had higher aerobic capacity and an increase in pentose phosphate pathway activity. The proteome profiles of eggs in the blastula stage were 131 proteins, of which 48 were significantly identified. The major protein was found to be phosvitin. The proteomes of underyearlings were represented by 2018 proteins, of which 49 were unique for the control and 39 for the experimental group. He–Ne laser irradiation had a strong effect on the contents of histone proteins.

## 1. Introduction

A promising approach in fish farming and aquaculture is the application of techniques from quantum biology. The application of low-level helium–neon (He–Ne) laser irradiation is well-known in a variety of biomedical and clinical tasks, such as anti-inflammation and anti-infection therapy for chronic wounds, after surgery and scalpel actions, in wound-healing procedures, in photobiomodulation, and in photostimulation therapy to prevent and treat muscle damage [1,2,3]. Applying He-Ne laser techniques in agriculture, such as in assisted-reproduction technologies, is widely discussed [4,5,6]. Low-intensity laser irradiation is known as a signaling factor for symmetrization and synchronization of morphogenesis, intensification of functional responses, acceleration of growth, reduction in phenotypic trait variations, and promotion of fish survival early in ontogeny.

Data on the effects of low-level laser irradiation on the oxidant/antioxidant balance during embryogenesis of fishes, embryo and larva morphology [7] and the functioning of membrane-bound enzymes, namely ATP hydrolases [8], suggest that He–Ne laser irradiation has a significant influence on the physiological conditions of cells [9]. The effect of photobiological stimulation is comparable to the action of various antioxidants, and quantum biology techniques in aquaculture can be listed among antioxidant prophylaxis methods. Even a single stimulatory treatment with laser irradiation during embryogenesis in fish increases their survival rates [6]. An important consideration, however, is the developmental stage in which low-level laser irradiation is applied. Previous studies showed, for instance, that in some fish species irradiation of eggs with He–Ne lasers during the gastrulation and embryonic motoric stages increased the survival of embryos and the hatching ratio of normal larvae, whereas irradiation during the organogenesis stage had a negative effect on the embryonic and early larval development of the fish [10,11]. Fish farmers often report *Saprolegnia* fungal infections on fertilized eggs [12], and treatment with laser irradiation during embryonic stages suppressed the fungus, raising the survival rate of the embryos.

The effects of low-level laser irradiation at the cellular and biochemical levels include activation of cell proliferation, collagen synthesis and growth factor production [13,14], and biogenesis of animal mitochondria [9,15]. For example, a 5-min treatment of loach embryos with a He–Ne laser in the first hours after fertilization influenced the shape, dimensions, and structure of mitochondrial cristae, and increased their number [9]. It has been demonstrated for various objects that He–Ne laser treatment notably activates a key enzyme in the electron transport chain—cytochrome c oxidase [14,16,17,18]. Activation of the electron transport chain increases the mitochondrial membrane electrical potential (DCmt), builds up the ATP pool, and, eventually, activates nucleic acid synthesis [19]. Studies have also proved the activity of lactate dehydrogenase LDH and creatine kinase during embryonic development in trout to be augmented by laser treatment during the eyed egg stage [20]. Besides cell energy activation associated with mitochondrial ATP synthesis, the laser biostimulation of development in animals, including fish, involves qualitative and quantitative changes in lipids and their fatty acid components, which are known to have an important role in the functioning of biological membranes.

It is safe to assume that laser treatment of fertilized fish eggs will promote the adaptive capacity, growth rate, and resilience of the artificially reared young released into the wild [6,12,21]. In view of this, an experiment was staged in this study to investigate the effects of irradiation with a He–Ne laser on the fertilized eggs of wild Atlantic salmon, Salmo salar. Radiation was applied during the early fission stage to examine changes in the biochemical status of hatched larvae. A comparative analysis was carried out covering the lipid profile (including fatty acids) and the activity of some key energy metabolism enzymes and proteomes in blastula-stage salmon eggs and in underyearlings (0+). The experiment was conducted in a hatchery where one of the main functions is compensatory weaning and release of salmon smolts to restore natural populations of the species in the North.

The multi-sided approach in the study was achieved by consolidating the efforts of ichthyologists, hydrobiologists, fish culture practitioners, and biochemists in assessing the growth, development, wellbeing, and quality of the reared young fish, as well as the effectiveness of the measures suggested to accelerate fish growth and enhance their natural resistance to conditions in the hatchery and during transition to the natural environment. Laser radiation strengthens the adaptive capacity of fish at early ontogenetic stages, thus offsetting (preventing, compensating, or attenuating) the effect of detrimental environmental factors. With its effect on eggs, photobiotic stimulation can be compared to the effect of various antioxidants, and quantum biology methods in aquaculture can be regarded as preventive antioxidant treatment. Overall, treatments with helium-neon laser radiation as well as infrared LED light, which can be classified as low-intensity impacts, promote the compensatory adaptation of fish organisms [21,22,23].

One must remark here that no such studies of equal scope as planned in this study have so far been implemented in North European Russia or globally. As a result, upgraded technology will be designed and suggested for application in practice, which is expected to significantly enhance the efficiency of artificial breeding of young salmon fish by reducing mortality, accelerating growth, and raising the resilience of the young released into the wild, and will contribute to the restoration of natural populations of valuable commercial fish species in waters of the Republic of Karelia and Kola Peninsula.

## 2. Materials and Methods

### 2.1. Experimental Design and Sample Collection

The experiment was conducted at the Vygsky fish hatchery (Belomorskij region, Russia, 64°25′ N and 34°28′ E). The incubation and rearing tanks had a flow-through supply of water from the Matkozhnenskoe reservoir (Nizhnij Vyg River).

**Figure 1:**
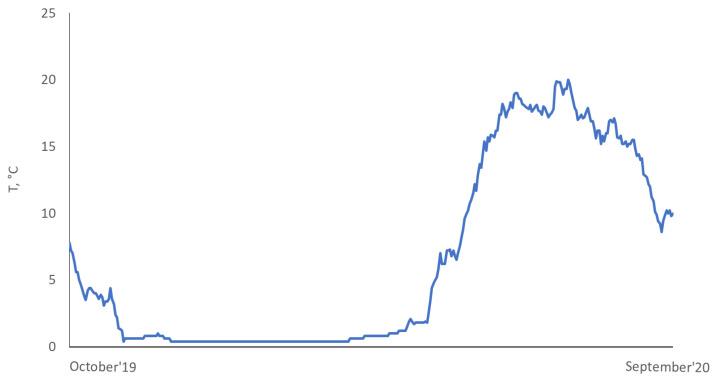
Water temperature from October 2019 to September 2020.

The eggs of the Atlantic salmon population of the Keret River (White Sea basin) were incubated in the control and experimental incubatory systems. Three trays were used: 3 trays in the control group and 3 trays in the experimental group, with about 7000 eggs in each tray of the incubator. The experiment started on 14 October 2019; the water temperature was 4.4 °C. The natural dynamic of temperature for the duration of the experiment is shown in Figure 1.

**Figure 2:**
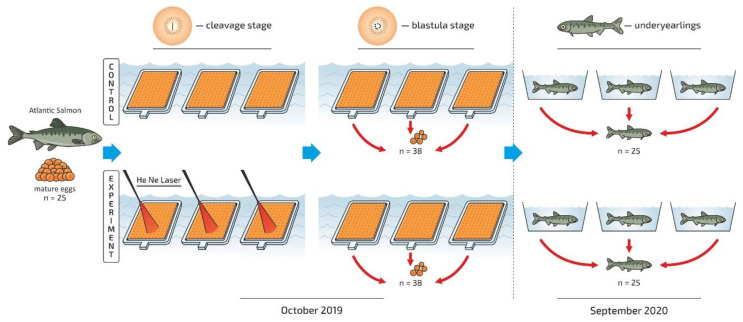
The general scheme of the experiment: the He-Ne irradiation was made at cleavage/early blastula stage. The samples were collected at progressive blastula and fingerlings stage in control and experimental group.

The effect of low-level irradiation with a He–Ne laser (0.63 μm, 1.5 × 10^−2^ J/cm^2^) on the lipid profile and the activity of energy metabolism enzymes, and the proteome at the blastula stage and in underyearlings (0+ age) after irradiation at the cleavage/early blastula was studied. It is known that the cleavage and early blastula stages of embryogenesis of salmon are when the cell has a high potential for differentiation. He–Ne laser irradiation of the experimental batch of eggs at the cleavage stage/early blastula was carried using the optimal dose interval (1.5 × 10^−2^ J/cm^2^) on 16 October 2019 (12.6 degree days). The source of coherent monochromatic irradiation was a continuous-mode He–Ne laser generator (GN-40). The device is characterized by the following technical parameters: the radiation wavelength is 0.63 μm, the radiation power is 40 mW, the spectral composition of the radiation is multimode, the diameter of the laser radiation beam at the level of γ = 0.9 is no more than 1.9 mm, the energy divergence of radiation is at the level of γ = 0.9—no more than 1.7 mrad—and power consumption is no more than 60 W. The scheme of the experiment is presented in Figure 2.

He–Ne laser irradiation of the developing eggs incubated in a tray at the cleavage stage/early blastula was performed. A pool of eggs from approximately 15 females was taken. The stage of eggs development was determined by ichthyologists at fishery. The total number of samples for the studied biochemical parameters is shown (Figure 2). Triplicate samples, three vials from each studied stage, were collected and prepared for analysis from certain lines of trays (treated/non-treated, triplicated variants).

A single hatching of salmon larvae was observed on 6 May 2020 (after 225.1 degree days), and the active hatching occurred on 12 May 2020. The process of “keeping” the larvae started on 18 May 2020, and the first exogenous feeding began on 22 May 2020. In June 2020, all larvae switched to endogenous feeding.

As soon as fry reached an average mass of 0.75 g, they were transferred from the incubation center (larval culture area) to the juvenile rearing tanks. The weight of the fry was 0.74 ± 0.03 g in the control group and 0.83 ± 0.02 g in the experimental group. The average number of fingerlings was 5400 per tank at the beginning of the experiment. The fish were kept in flow-in tanks (2 m × 2 m) and with a water volume of 720 L. All rearing conditions, such as fish-holding density, feed and feeding regime, preventive measures, and care of the tanks, remained the same for all studied tanks. The flow velocity was 40 L min^−1^. Water temperature regime was natural (Figure 1). The fish were fed with commercial feed according to the recommendations of the fish hatchery, and this depended on water temperature fluctuations. The daily ration for the underyearlings was 3.1–3.3% in July, 3.3–2.4% in August, and 2.2–1.2% in September. Automatic feeders were used. Commercial feed (BioMarInicio^®^ 917, BioMar, Aarhus, Denmark), which contained 57–58% crude protein, 14–18% crude lipid, and 8–10% carbohydrates, depending on pellet size, was used. The feeding regime was organized according to the general rules and recommendations of the hatchery. It was based on water temperature and sustainable feeding practices relating to hatchery-reared fish.

The underyearlings were weighed several times every month. Data on fish weight were obtained from the results of the repeated weighing (five times) of 100 individuals together, as described previously [24].

Samples for biochemical and molecular genetic analysis and proteomic analysis were taken at the early-blastula stage (24 October 2019, 41.5 degree days) and at the stage of fry underyearlings, 0+ age (17 September 2020).

For lipid profiling, 15 individual eggs at blastula stage without laser irradiation were collected from 3 incubation trays (the control group), and 15 irradiated eggs at blastula stage as part of the laser experiment (LE) were collected from 3 other incubation trays. Fifteen underyearlings from each rearing tank—control and LE—were collected individually. Samples were fixed in chloroform: methanol, 2:1 (*v:v*). In addition, 15 mature eggs were collected at the beginning of the experiment.

The samples for the analyses of enzyme activity and proteome were collected on the same dates, fixed in liquid nitrogen, and stored at −80 °C. There were 15 eggs per group (control and LE) at the early blastula stage for enzyme activity analyses and 5 eggs per group for proteomics. For underyearlings, the epaxial (white) muscle samples were collected (*n* = 10 per group for enzyme analyses and *n* = 5 per group for proteomic). Twenty-five mature eggs were taken at the beginning of the experiment for analysis of enzyme activity. A description of the eggs and fish taken for analysis is provided in Table 1.

### 2.2. Lipid Extraction and Lipid Classes Analysis

Individual samples of mature eggs, eggs at blastula stage and underyearlings from control and LE were homogenized in glass vials in chloroform/methanol (2:1, *v*/*v*) solution (10 mL per 1–3 g wet weight). The total lipids (TLs) were extracted using the Folch method [25]. The procedure of the lipid extraction is presented in our previous study [24].

Qualitative and quantitative determination of individual lipid classes as total phospholipids (PL), mono-, di-, triacyclglycerols (MAG, DAG, TAG respectively), cholesterol (Chol), sterol esters (mainly cholesterol esters) and non-esterified fatty acids (NEFA) was carried out using the high-performance thin-layer chromatography (HPTLC) using a equipment complex produced by CAMAG (CAMAG, Switzerland) as a semi-automatic Linomat 5 applicator (CAMAG, Switzerland) to apply microvolumes of the sample on ultrapure glass-based plates—HPTLC Silicagel 60 F_254_ Premium Purity (Merck, Germany). Then the separation of individual lipid classes was carried out using an ADC2 chromatographic chamber (CAMAG, Switzerland), the derivatization was made using Derivatizer (CAMAG, Switzerland). Qualitative and quantitative determination of lipid components was carried out in the chamber of a TLC Scanner 4 densitometer (CAMAG, Switzerland).The HPTLC method description given in Reference [24]. The identification of lipid classes was carried out according to the standards of the respective studied components (among sterol esters the cholesterol esters standards were used) (Sigma-Aldrich, St. Louis, MO, USA; Avanti Polar Lipids, Inc., Alabaster, AL, USA). The identification of individual lipid classes was carried out according to the standards of the respective studied components (“Sigma-Aldrich”, USA), taking into account the correspondence of the Rf-values.

### 2.3. Certain Phospholipid Fractions Analysis by HPLC

The analysis procedure was the same as described in Reference [26]. The spectra of individual phospholipid fractions were determined by high-performance liquid chromatography (HPLC) using Aquilon Stayer HPLC (Aquilon LLC, Moscow, Russia) using a Nucleosil 100-7 C18 HPLC column with a acetonitrile:hexane:methanol:phosphorus acid (918:30:30:17.5, by volume) mobile phase, the rate of moving was 540 mkl/h, the volume of the injected sample was 5 µL, the injector Rheodyne 7725i. The detection was performed using a spectrophotometer (UV light, 206 nm), the method was isocratic. Samples were manually injected using Rheodyne Valco Beckman and SSI Valves syringes (Hamilton, FL, USA). Phospholipids standards (Sigma Aldrich, St. Louis, MO, USA) were used for the identification and quantification of the phospholipid compounds in the sample. We identified six phospholipids: phosphatidylserine (PS), phosphatidylethanolamine (PE), phosphatidylinositol (PI), phosphatidylcholine (PC), lysophosphatidylcholine (LysoPC) and sphingomyelin (SM).

### 2.4. Fatty Acid Analysis

Qualitative and quantitative fatty acids (FAs) profile of the TLs was analyzed by gas liquid chromatography (GC) with a flame-ionized detector (FID) and mass-detector (MS). FAMEs were separated on a GC with mono-quadrupole mass-selective detector “Maestro-αMS” (Saitegra, Russia) for identification FAs constituents. The separation of FAs was carried out for 120 minutes in isothermal configuration (200 °C) on a Zebron ZB-FFAP capillary column (Phenomenex, Torrance, CA, USA) using helium as a mobile phase. The SIM/SCAN mode: SIM mode for searching for FAs according to the analytical standards – Supelco 37, Bacterial Acid Methyl Ester (BAME) Mix and PUFA №1 Marine source (all Sigma Aldrich, USA); SCAN mode was used for searching and identification unique FAs with scan parameters 50 to 400 m/z. The data were analyzed using “Maestro Analytic v. 1.025” software with NIST library. Next, after qualitative identification FA with GC-MS, the quantitative determination was carried out using GC-FID. FAMEs were separated on a “Chromatek-Crystall-5000.2” gas chromatograph with a flame-ionization detector (FID) and an automatic liquid dispenser (Chromatek, Yoshkar-Ola, Russia). The separation of FAs was carried out for 120 minutes in isothermal configuration (200 °C) on a Zebron ZB-FFAP capillary column (Phenomenex, Torrance, CA, USA) using nitrogen as a mobile phase. Chromatek-Analytik-5000.2 software “Chromatek Analytic V. 3.0.298.1” (Chromatek, Yoshkar-Ola, Russia), the analytical procedure is described in Reference [26]. All GC parameters were identical between GC-MS and GC-FID except mobile phase (helium and nitrogen, respectively). 

### 2.5. Enzyme Analyses

The activities of the enzymes were determined for eggs and the muscle samples and were assayed spectrophotometrically (CLARIOSTAR, BMG Labtech, Ortenberg, Germany). Tissue samples were homogenised in a 0.05 M Tris-HCl buffer (pH 7.5) by Tissue Lyser (Qiagen, Hilden, Germany). The activity of cytochrome c oxidase (COX, E.C.1.9.3.1) was measured based on the oxidation of cytochrome c and was determined at 550 nm [27]. Activities of lactate dehydrogenase (LDH, E.C.1.1.1.27, [28]), glucose-6-phosphate dehydrogenase (G6PDH, E.C.1.1.1.49, [29]) and glycero-3-phosphate dehydrogenase (GPDH, E.C 1.1.1.8 [29]) were assayed at 340 nm. One unit of enzyme activity was defined as the amount of enzyme necessary to elicit a change in absorbance of 1.0 unit/min (1.0-cm light path). The extinction coefficient for NADH + H+ or NADPH was 6.22 cm^−1^ µmol^−1^. Aldolase (E.C.4.1.2.13, [30]) activity was determined at 535 nm based on the colorimetric method in which the dinitrophenylhydrazones of the free trioses are determined. The enzyme activities were expressed as units, where one activity unit equalled the formation of 1 nmol of the product per minute, per mg of protein. The total protein concentration was determined using the Bradford method [31].

### 2.6. Proteome Analysis

Three eggs at the blastula stage were pooled for each control group (0.122 g) and experimental group (0.174 g) and then destroyed in 300 μL of 50 mM triethylammonium bicarbonate with an ultrasonic probe in cold conditions for 1 min. The remainder of the undestructed shell was precipitated with 4000 *g* for 2 min. After that, the protein precipitation was performed using the methanol-chloroform method. The supernatant was mixed in a ratio of 300 µL with 1200 µL of methanol well mixed, then 300 µL of chloroform also well mixed and water in a volume of 900 µL followed by stirring. Pooled samples were centrifuged at 14,000 *g* for 2 min. The upper phase was withdrawn, and the precipitate was redissolved in 900 µL of methanol followed by stirring and centrifugation at 14,000 *g* for 2 min. The dried precipitate after extraction with methanol was redissolved with 2% sodium deoxycholate. Each sample protein concentration was measured using a Pierce™ BCA Protein Assay Kit (Pierce, Rockford, IL, USA) in accordance with the manufacturer’s recommendations [32]. A total protein amount of 100 µg of each pooled sample was used for in-solution digestion with trypsin. For reduction and alkylation of disulfide bonds, the samples were brought to a volume of 50 μL by buffer containing 50 mM TEAB (pH = 8.5), incubated in the presence of 4 mM tris(2-carboxyethyl)phosphine (TCEP) and 6.2 mM 2-chloroacetamide (CAA) at 80 °C for 30 min. Tryptic digestion was carried out overnight at 37 °C with trypsin (Sequencing Grade Modified, Promega, Madison, WI, USA) with protein/trypsin ratio 1:50 in 8 mM tetraethylammonium bicarbonate buffer (pH 8.5). To stop the hydrolysis, formic acid was added to a final concentration of 5%, followed by centrifugation at 11,000 *g* for 15 min. The peptides were dried in a vacuum concentrator and dissolved in 20 mL of 5% formic acid for subsequent MS analysis.

Five livers of underyearlings were pooled for each control group and experimental group. Then the pooled samples were lysed using ice-cold buffer (150 µL) containing 3% sodium deoxycholate, 2.5 mM EDTA, 75 mM Tris-HCl (all Sigma-Aldrich, St. Louis, MO, USA), pH 8.5 and protease inhibitors cOmplete™ (Roche, Basel, Switzerland) with subsequent ultrasonication using the Bandelin Sonopuls probe (“BANDELIN electronic GmbH & Co. KG”, Berlin, Germany). The cell lysates were centrifuged for 15 min at 5000× *g* using Eppendorf 5424R centrifuge. The supernatants were collected, and the pellets were dissolved in 100 µL of lysis buffer, and then subjected to the second round of protein solubilization as described above. The sample protein concentration was measured using a Pierce™ BCA Protein Assay Kit (Pierce, Rockford, IL, USA). Protein digestion was performed according to the protocol described in detail by Zgoda et al. [33]. Briefly, the protein sample (50 µg) was transferred into a clean tube and denaturation solution (5 M urea, 1% sodium deoxycholate, in a 50 mM triethylammonium bicarbonate buffer (TEAB) containing 20 mM dithiothreitol (DTT) (all Sigma-Aldrich, St. Louis, MO, USA) 20 mM DTT) in volume of 20 µL was added to make the final concentration of total protein close to 5 mg/mL. Then the samples were heated for 60 min at 42 °C and, after cooling at room temperature, 25 µL of 15 mM 2-iodoacetamide in 50 mM TEAB was added. The alkylation reaction continued for 30 min at room temperature and the sample was then diluted up to 120 µL by 50 mM TEAB to decrease the final concentration of denaturation buffer compounds and dilute the final protein concentration close to 0.5 mg/mL. Trypsin (1 µg) was added to samples and incubated overnight at 37 ◦C. The hydrolysis was stopped by adding formic acid (to a final concentration of 5%). Samples were centrifuged for 10 min at 10 °C at 12,000 ×*g* to sediment deoxycholic acid. The peptides were dried in a vacuum concentrator and dissolved in 20 mL of 5% formic acid for subsequent MS analysis.

The peptides were separated with high-performance liquid chromatography (HPLC, Ultimate 3000 Nano LC System, Thermo Scientific, Rockwell, IL, USA) in a 15 cm long C18 column (Acclaim^®^ PepMap™ RSLC inner diameter of 75 µm, Thermo Fisher Scientific, Rockwell, IL, USA). Prior the separation, one microgram of peptides in a volume of 1 μl was loaded directly onto the HPLC column at a flow rate of 0.3 μL/min for 12 min in an isocratic mode of Mobile Phase C (2% acetonitrile, 0.1% formic acid). The peptides were eluted with a gradient of buffer B (80% acetonitrile, 0.1% formic acid) at a flow rate of 0.3 µL/min. The total run time was 90 min, which included initial 10 min of column equilibration to 2% buffer B, then gradient from 2 to 35% of buffer B over 68 min, then 2 min to reach 99% of buffer B, flushing 2 min with 99% of buffer B, and a linear decrease in the concentration of buffer B to the original 2% in 3 min and 5 min re-equilibration.

MS analysis of the samples was performed in triplicate with a Q Exactive HF-X mass spectrometer (Q Exactive HF-X Hybrid Quadrupole-Orbitrap^TM^ Mass spectrometer, Thermo Fisher Scientific, Rockwell, IL, USA) in positive ionization mode using an nESI ion source. The temperature of the capillary was 240 °C, and the voltage at the emitter was 2.1 kV. Mass spectra were acquired at a resolution of 120,000 (MS) in a range of 300−1500 m/z. Tandem mass spectra of fragments were acquired at a resolution of 15,000 (MS/MS) in the range from 100 m/z to m/z value determined by a charge state of the precursor, but no more than 2000 m/z. Isolation of precursor ions was performed in ±1 Da window. Up to 40 ions was set as the maximum number of ions allowed for isolation in MS2 mode. For tandem scanning, only ions with a charge state from z = 2+ to z = 6+ were taken. The maximum integration time was 50 ms and 110 ms for precursor and fragment ions, respectively. AGC target for precursor and fragment ions were set to 1 × 106 and 2 × 105, respectively. An isolation intensity threshold of 50,000 counts was determined for precursor selection, and the top 20 precursors were chosen for fragmentation with high-energy collisional dissociation (HCD) at 29 NCE. All measured precursors were dynamically excluded from triggering a subsequent MS/MS for 70 s.

The mass spectra raw files were loaded into the MaxQuant v.1.6.4.3 program [34]. The searches were performed using the Andromeda algorithm (built into MaxQuant) using the Salmonidae database provided by UniProt (September 2020). The following search parameters were used: enzyme specificity was set to trypsin two missed cleavages were allowed. Carbamidomethylation of cysteines was set as fixed modification and methionine oxidation and N-terminal proteins acetylation was set as variable modification for the peptide search. The mass tolerance for precursor ions was 4.5 ppm; the mass tolerance for fragment ions was 20 ppm. Peptide Spectrum Matches (PSMs), peptides, and proteins were validated at a 1.0% false discovery rate (FDR), estimated using the decoy hit distribution. Proteins were considered to be significantly identified if at least two peptides were found for them. Protein quantification was based on the iBAQ.

### 2.7. Statistical Analysis

Lipid analysis: To perform statistical analysis, the free R-programming language (v. 3.6.1.) in IDL RStudio (v. 1.2.5019) with basic packages and additional “readxl” (v. 1.3.1), “tidyverse” (v. 1.3.0), “corrgram” (v. 1.13), “factoextra” (v. 1.0.6), “cowplot” (v. 1.1.1) packages was used. Descriptive statistics results are presented in the “M ± SE” format. Statistical significance differences between individual lipids and fatty acids were tested by non-parametric test, the Wilcoxon-Mann-Whitney test [35]. Statistical significance was set at *p* ≤ 0.05.

Enzyme analysis: Data were analyzed using the Kruskal-Wallis test. Differences between groups were evaluated by Mann-Whitney *U* test. All results were considered significant at *p* < 0.05. All data are presented as the means ± SE.

Proteomic analysis: R-programming language (v. 3.6.1.) was used for statistical analysis, modeling and visualization data with additional packages “readxl” (v. 1.3.1) “tidyverse” (v. 1.3.0), “factoextra” (v. 1.0.6), “gplots” (v. 3.0.3), “corrgram” (v. 1.13), “igraph” (v. 1.2.6), “tm” (v. 0.7–8), “wordcloud” (v. 2.6), “cowplot” (v. 1.1.1), “randomForest” (v. 4.6–14). The words cloud was formed by requesting the frequency of occurrence (at least 3 times) of individual words in the entire list of proteins. At the same time, punctuation and identifiers were removed from the analysis. iBAQ values were Z-scores of normalized for further statistical analysis. Hierarchical clustering was carried out in the Euclidean coordinate system. Correlation analysis was carried out according by Spearman test. Machine learning was performed using classification algorithm “random forest”.

## 3. Results

### 3.1. Growth and Development of Fish during the Experiment

The results of ichthyological studies and fish-breeding observations of the growth and development of Atlantic salmon under artificial rearing conditions after laser irradiation of fertilized eggs at the stage of cleavage/early blastula showed that during the incubation period (from November to April), the loss of eggs (% of mortality) in the experimental group was 3.5% compared with 8.2% in the control group. Hatched larvae in the experimental group, in the process of further rearing (July–October), had increased weight growth and low mortality rates compared to those in the control group. In July, the average weight of the larva from eggs exposed to laser irradiation was 2.6 g, and the loss was 0.07%. In the control group, the average weight declined by 1.9 g and the mortality rate was 0.15%. For underyearlings average weight and loss were as follows: In August, 5.3 g and 0.3% for the experimental group, and 4.3 g and 0.7% for the control group; In September, 8 g and 0.5% for the experimental group, and 7.1 g and 1.1% for the control group; In October, 9.3 g and 1.2% for the experimental group, and 8.7 g and 1.5% for the control group.

### 3.2. Lipid Profile and the Activity of Energetic Metabolism Enzymes in Mature Eggs (Unfertilized)

**Figure 3:**
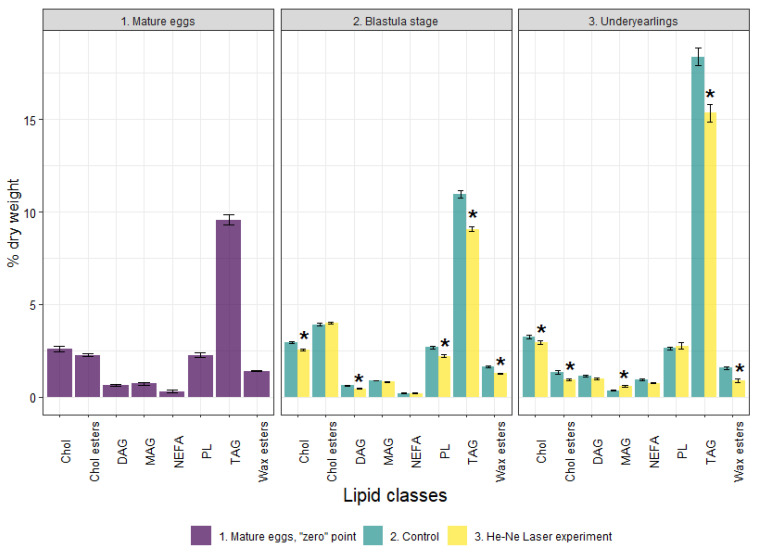
Lipid profile in mature (unfertilized) eggs, at the blastula stage and in underyearlings of Atlantic salmon in control and experiment. *—significantly different (*p* < 0.05) between groups. Chol—cholesterol, Chol esters—cholesterol esters, DAG—diacylglycerols, MAG—monoacylglycerols, NEFA—non-esterified fatty acids, PL—total phospholipids, TAG—triacylglycerols.

**Figure 4:**
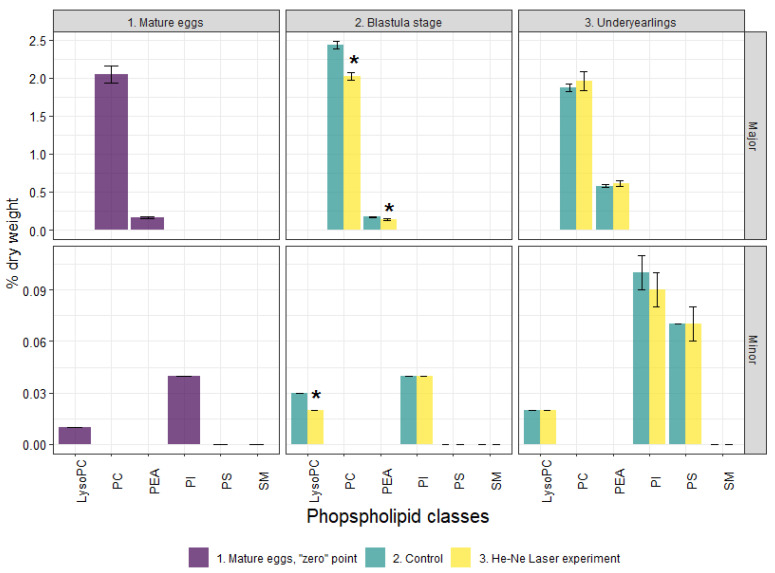
Phospholipid profile in mature (unfertilized) eggs, at the blastula stage and in underyearlings of Atlantic salmon in control and experiment. *—significantly different (*p* < 0.05) between groups. LysoPC—lysophosphatidylcholine, PC—phosphatidylcholine, PEA—phosphatidylethanolamine, PI—phosphatidylinositol, PS—phosphatidylserine, SM—sphingomyelin.

The content of TLs in mature eggs was 19.78 % dry weight with the dominance of neutral lipids, also known as storage lipids—TAG (9.56% dry weight)—and Chol ester accounted for 2.27% (Figure 3). Waxes accounted for 1.4% to ensure the buoyancy and thermal insulation of eggs. The polar lipids—total PLs and the studied PL fractions—and cholesterol, both considered as main membrane components, were 2.27% and 2.62% dry weight, respectively (Figure 3 and Figure 4). Minor lipid classes such as MAG and DAG—intermediated biomolecules of metabolic processes of storage and membrane lipids—accounted for 0.72% and 0.64%, respectively. Non-esterified fatty acids were 0.31% dry weight.

**Figure 5:**
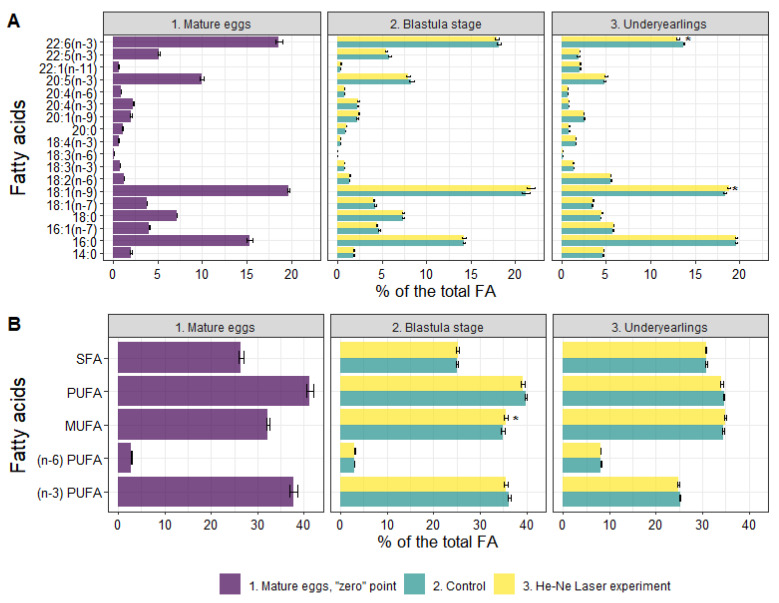
Fatty acid profile in mature (unfertilized) eggs, in blastula stage and in underyearlings of Atlantic salmon in control and experiment. *—significantly different (*p* < 0.05) between groups. SFA—saturated fatty acids, PUFA—polyunsaturated fatty acids, MUFA—monounsaturated fatty acids.

Polyunsaturated fatty acids were prominent in mature eggs of salmon—41.28% of the sum of FAs due to n-3 PUFAs (37.74%) (Figure 5). Among n-3 PUFAs, the essential docosahexaenoic (DHA, 22:6n-3) dominated at 18.63%. The content of MUFAs and SFAs was 32.27% and 26.42%, respectively, among which 18:1n-9 oleic FA and 16:0 palmitic FA (19.68% and 15.35%, respectively) were prevalent. Certain metabolic FA indexes characteristic of mature eggs of spawners of salmon (wild population) are shown in Table 2.

The activity of LDH and aldolase enzymes in unfertilized eggs was analyzed (Table 3). The activities of the enzymes COX, 1GPDH, and G6PDH were too small to determine. According to the calculation of the coefficient of variation, the sample was homogeneous, but there was a high variability of activity between eggs of 27.59% for LDH and 30.33% for aldolase. This indicated a heterogeneity in the level of anaerobic metabolism at the stage of unfertilized eggs. 

### 3.3. Lipid Profile, the Activity of Energetic Metabolism Enzymes and Proteome at the Blastula Stage

The content of TLs decreased significantly in the experimental group compared to the control group. The total PLs included PC, PEA and LysoPEA, TAG, DAG, Chol, and Chol esters. Along with this, the Chol/total PL and Chol esters/Chol ratios increased (1.16 and 1.55-fold in the experimental group compared with 1.10 and 1.33-fold in the control group. No significant differences were found in FA profiles at the blastula stage between the groups (Figure 5). 

At the blastula stage, high variability in the activity of these enzymes LDH and aldolase between eggs in both the experimental and control groups was maintained. It amounted to 21.75% (control) and 30.7% (experiment) for LDH and 28.59% (control) and 28.76% (experiment) for aldolase. For yearlings, the variability of these enzymes in the muscles was low (Table 3). The laser-irradiated embryos differed from the control group in the level of COX activity, which was 1.5 times higher (*p* < 0.05) (Table 3). At the same time, the LDH/COX ratio (the ratio of anaerobic to aerobic ATP synthesis) in the experimental group was 2 times lower than that in the control (*p* <  0.05) (Table 3). In the experiment al group, the level of G6PDH activity was higher by 1.3 times (*p* < 0.05) and 1GPDH by 1.5 times (*p* < 0.05) (Table 3) compared with the control group. There were no differences in the activity of aldolase in the two groups of embryos (Table 3).

General characteristics of the proteome profile of fertilized eggs at the blastula stage of Atlantic salmon in both groups were determined. No qualitative difference in the proteome of developing eggs at the blastula stage was detected between the groups. The analysis of the high-throughput proteomics of the salmon eggs led to an initial identification of 131 proteins, of which 48 were significantly identified using MaxQuant and the UniProt database, limited by species from the Salmonidae family.

**Figure 6:**
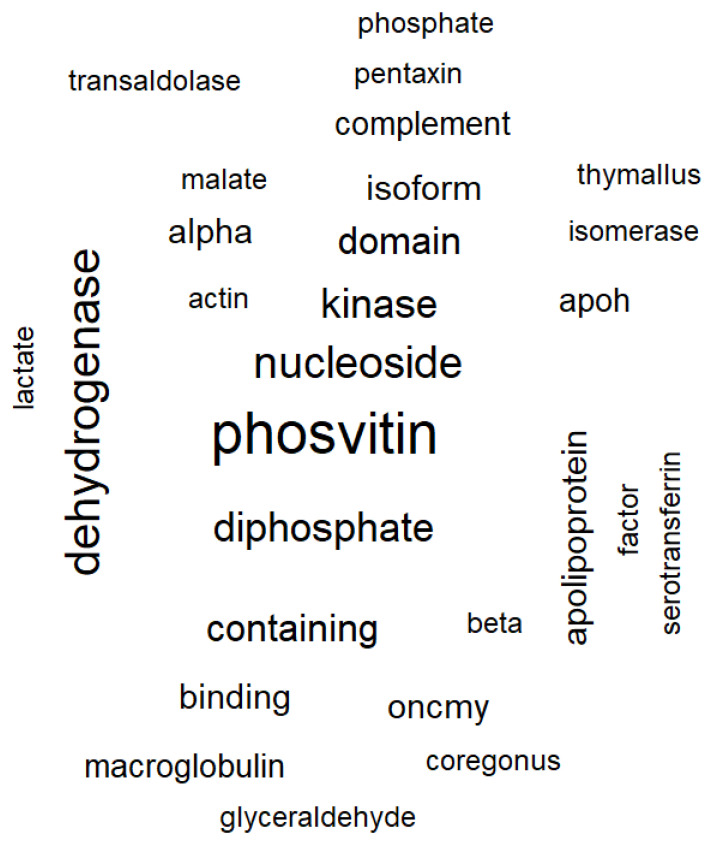
Words cloud by frequency of occurrence among identified proteins. Note: the closer to the center and the larger the font, the more often the word appears. The minimum frequency of occurrence of a word is 3 times.

**Figure 7:**
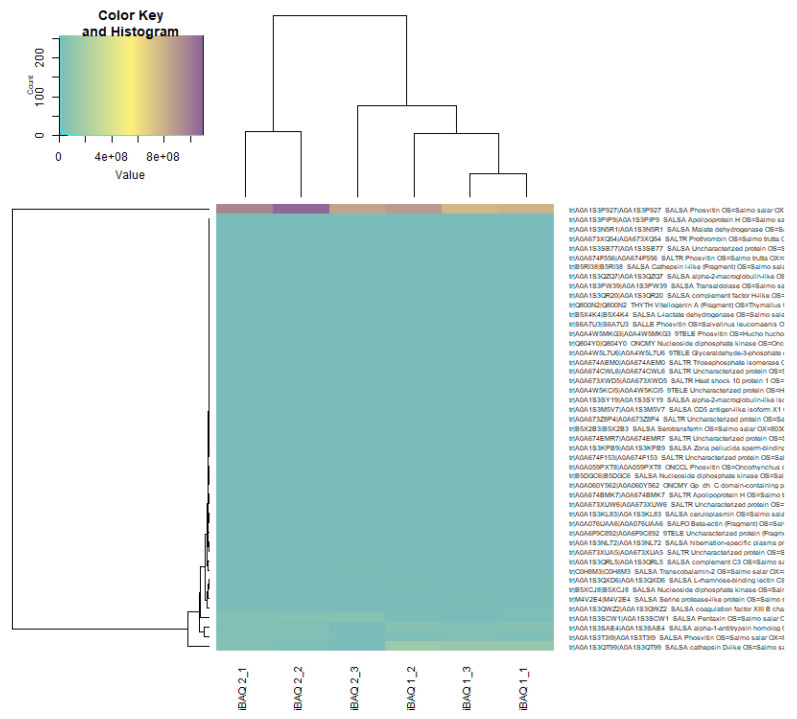
Heatmap of raw LFQ data in proteome of Atlantic salmon eggs (*Salmo salar* L.) for control and experimental groups. Note: iBAQ1_X is a control group, iBAQ2_X is an experimental group, where “X” is a serial number. Quantities are presented in gradient format (legend top left). The upper dendrogram is the hierarchical clustering for the experimental group, the left dendrogram is for the identified proteins.

The R programming language was used for exploratory data analysis (EDA) and statistical data analysis. The results showed that the dominant protein (in quantitative terms) was phosvitin, the main phosphoprotein of eggs (Figure 6 and Figure 7). The test of the frequency of occurrence of individual words in the list of identified proteins established that the most common group of proteins in Atlantic salmon eggs are dehydrogenases (glyceraldehyde-3-phosphate dehydrogenase, lactate dehydrogenase, and malate dehydrogenase), followed by nucleosides, diphosphates, and kinases represented by one protein (nucleoside diphosphate kinase).

**Figure 8:**
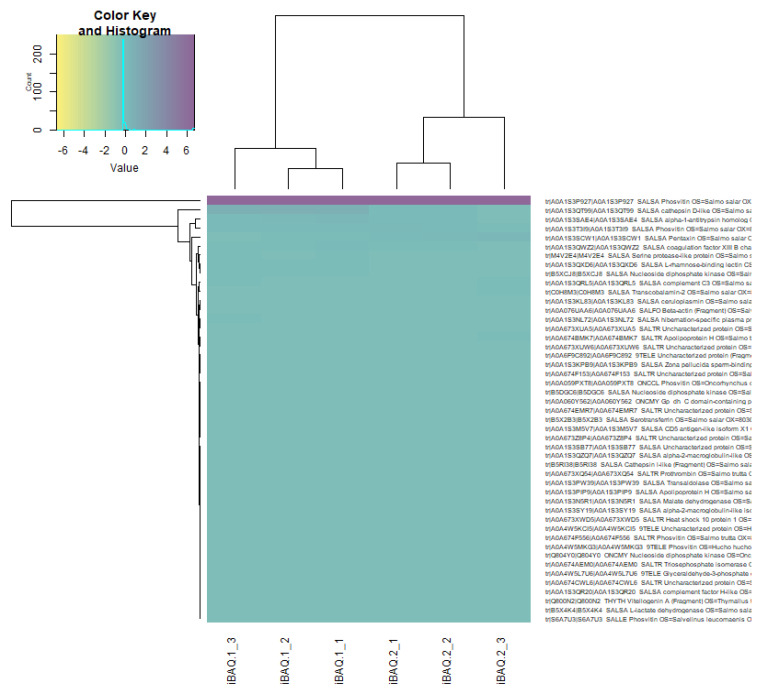
Heatmap LFQ Z-scores of normalized data in proteome of Atlantic salmon eggs (*Salmo salar* L.) for control and experimental groups. Note: iBAQ1_X is a control group, iBAQ2_X is an experimental group, where “X” is a serial number. Quantities are presented in gradient format (legend top left). The upper dendrogram is the hierarchical clustering for the experimental group, the left dendrogram is for the identified proteins.

**Figure 9:**
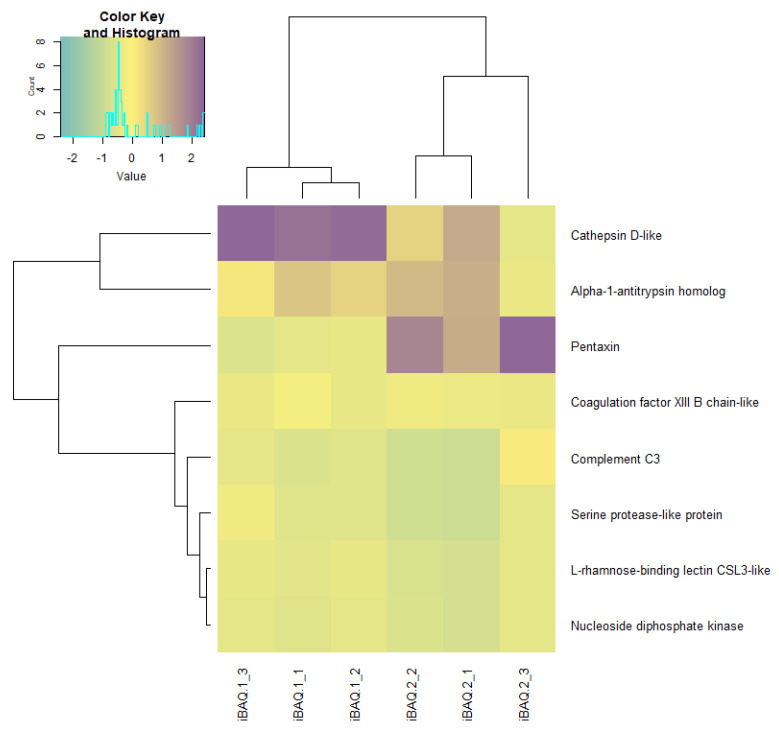
Heatmap for 9 selected proteins of Atlantic salmon eggs (*Salmo salar* L.) for the control and experimental groups. Note: Phosvitin has been removed from the graph to exclude high noise levels. IBAQ1_X—control group, iBAQ2_X—experimental group, where X is an ordinal number. Quantities are presented in gradient format (legend top left). The upper dendrogram is the cluster analysis for the experimental group, the left dendrogram is for the identified proteins.

Quantitative differences in proteome at the blastula stage of Atlantic salmon in the control group and in the experimental group were observed. Using the Z-scores of normalized data, nine proteins were identified, the content of which varied between the control and experimental groups (Figure 8, Table 4). In the experimental group of salmon eggs, the concentration of pentaxin decreased, and the content of cathepsin D and alpha-1-antitrypsin homolog increased (Figure 9). Based on the cluster analysis for control and experimental groups in terms of changes in the content of all 48 proteins (Figure 8, top) and for the nine selected proteins (Figure 9, top), we can assume that the effect of laser irradiation on salmon eggs at the blastula stage affects metabolic processes involving these proteins.

**Figure 10:**
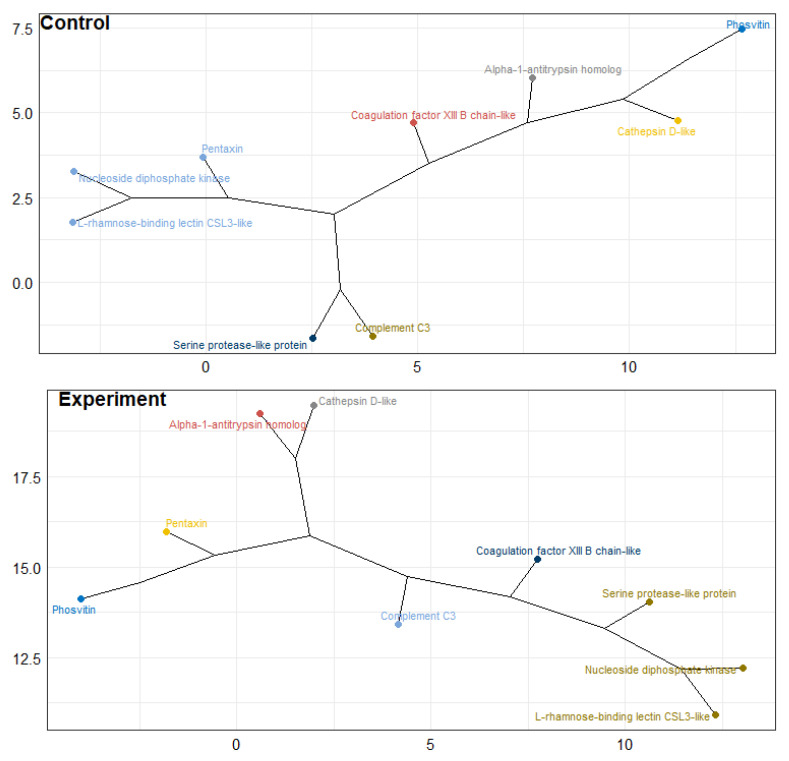
Hierarchical clustering visualizing as a “phylogenetic tree dendrogram” for control and experimental groups of Atlantic salmon eggs (*Salmo salar* L.). Note: 7 clusters are found out.

The “random forest” machine learning algorithm was used for the nine selected proteins from the control and experimental groups of salmon eggs. The analysis showed that the “strongest” classifiers between groups (factor-induced differences) were L-rhamnose-binding lectin CSL3-like, pentaxin, cathepsin D, serine protease-like protein, and nucleoside diphosphate kinase according to the mean decrease in Gini coefficient (MDG) (Table 5). This result was confirmed by a strong change in the concentration of the five selected proteins in comparison with others on the hierarchical clustering (visualized as a “phylogenetic tree dendrogram”) of the studied groups of salmon eggs (the dendrogram was based on Euclidean distances between points) (Figure 10).

Further, during the cluster analysis of nine proteins, an opposite and synchronous change in the content of nucleoside diphosphate kinase and L-rhamnose-binding lectin CSL3-like was noted with the saving of the initial cluster. Analysis confirmed a significant correlation between these proteins (r = 0.98). In addition, correlation analysis showed a relationship between these proteins and cathepsin D (r = 0.93 and 0.97, respectively), but an inverse correlation was noted between cathepsin D-like and pentaxin (r = −0.96). At the same time, a negative correlation with Serine protease-like protein also was noted for pentaxin (r = −0.95).

### 3.4. Lipid Profile and the Activity of Energetic Metabolism Enzymes and Proteome in Underyearlings of Atlantic Salmon

Compared to the control group, the content of TLs significantly decreased in the experimental group due to DAG, TAG, Chol, Chol esters, NEFAs, and waxes in the underyearlings. No significant differences were detected in the content of total PLs and certain fractions. It was found that the Chol/PL, TAG/PL and Chol esters/Chol ratios decreased (by 1.14, 1.23, and 1.31-times) in fish in the experimental group compared to the control group. The total Pl + Chol/TAG + Chol esters + waxes ratio (membrane lipids/storage lipids ratio) was higher in fish in the experimental group than in the control group (0.33 vs. 0.28). The FA profile in underyearlings was characterized by an almost equal content of PUFAs and MUFAs. Among certain FAs, only the content of DHA significantly decreased and 18:1n-9 increased in fish in the experimental group compared with the control group (Figure 5). It is interesting that the 20:5(n-3)/18:3(n-3) ratio was significantly higher and the 22:6(n-3)/20:5(n-3) ratio was lower in underyearlings in the experimental group compared with the control group (Table 2).

The yearlings in September did not differ in the levels of COX, LDH, and aldolase activities (Table 3).

**Figure 11:**
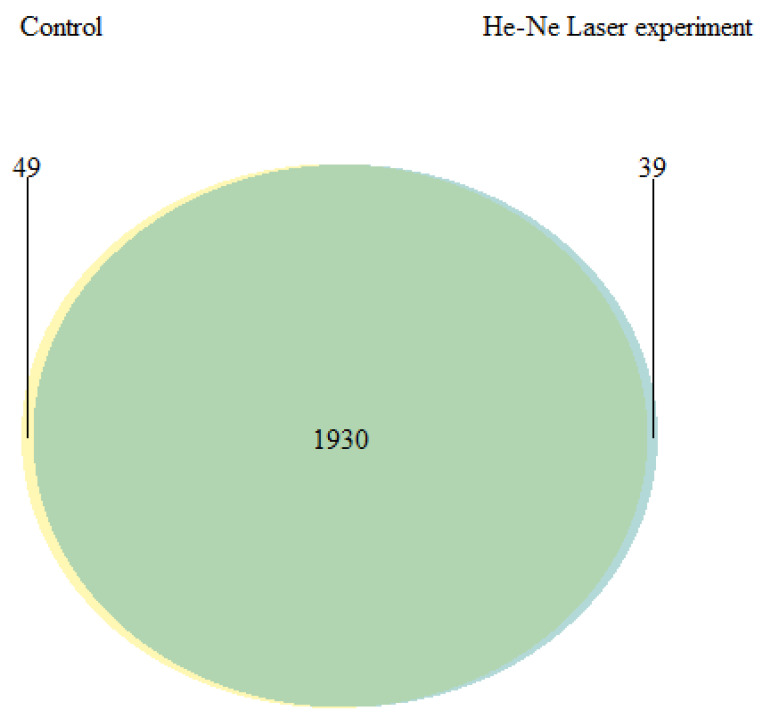
Venn diagram for the proteome of underyearlings of Atlantic salmon (*Salmo salar* L.) for the control and experimental groups.

Qualitative and quantitative differences were established in the proteome of underyearlings of Atlantic salmon from the control and experimental groups. Mass spectrometric analysis of the proteome of underyearlings revealed 2018 proteins, of which 49 were unique for the control group and 39 for the experimental group (Figure 11, Table 6). Proteins were identified using the MaxQuant program and the UniProt database.

**Figure 12:**
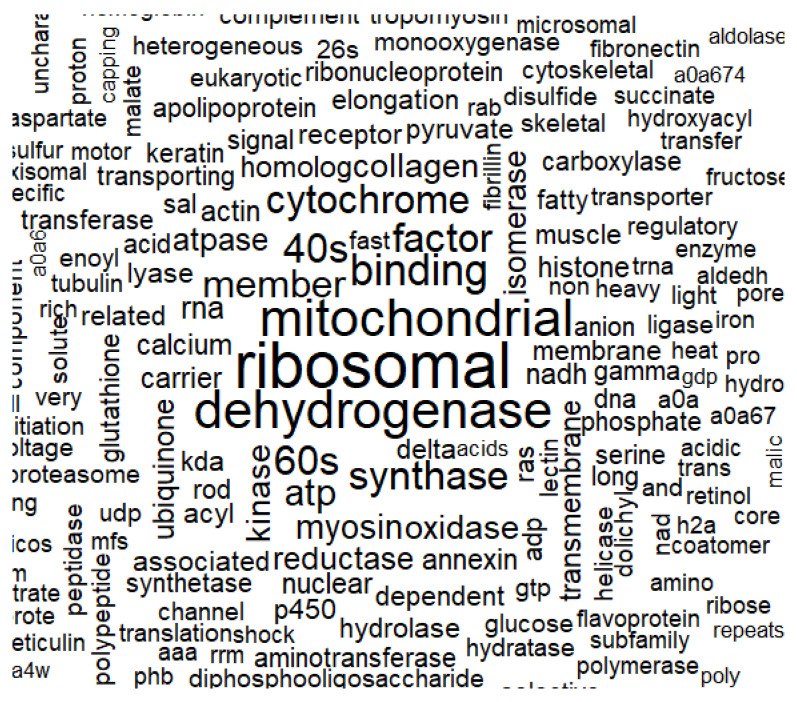
Words cloud by frequency of occurrence among identified proteins. Note: the closer to the center and the larger the font, the more often the word appears. The minimum frequency of occurrence of a word is 10 times.

The R programming language was used for exploratory data analysis (EDA) and statistical data analysis. The results showed that the most common proteoform in underyearlings was the ribosomal protein (which occurred 93 times in the experimental group and 94 times in the control group). Common protein families (in terms of frequency of occurrence) were mitochondrial proteins, dehydrogenases, synthases, cytochromes, myosine, and oxidases (Figure 12).

**Figure 13:**
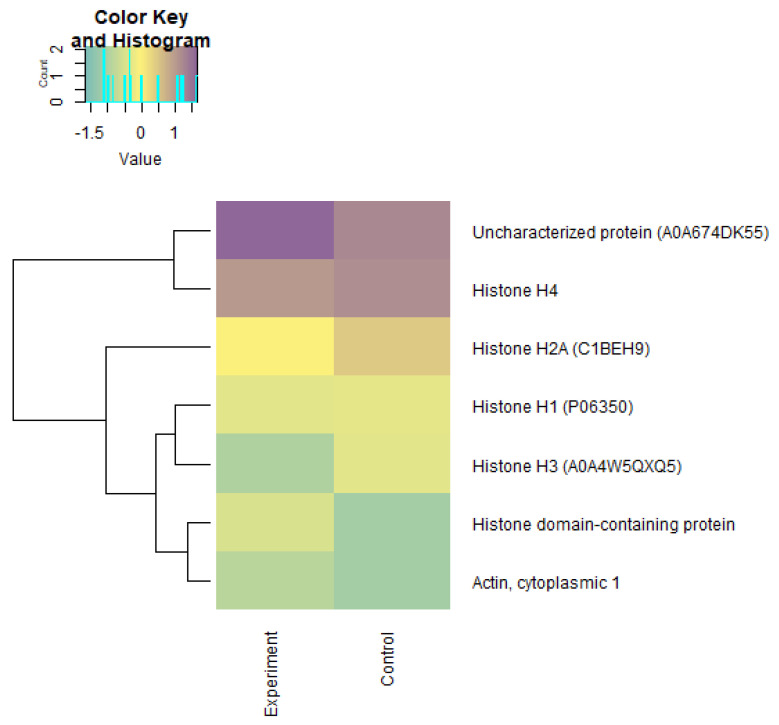
Heatmap LFQ Z-scores of normalized data of a “high concentration” proteome (group 1) in underyearlings of Atlantic salmon (*Salmo salar* L.) for control and experimental groups.

**Figure 14:**
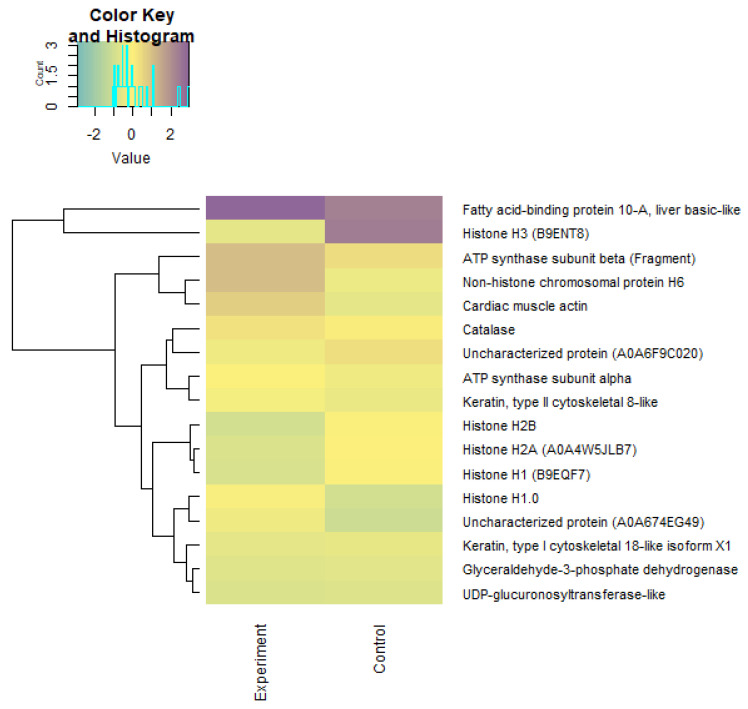
Heatmap LFQ Z-scores of normalized data of a “mean concentration” proteome (group 2) in underyearlings of Atlantic salmon (*Salmo salar* L.) for control and experimental groups.

**Figure 15:**
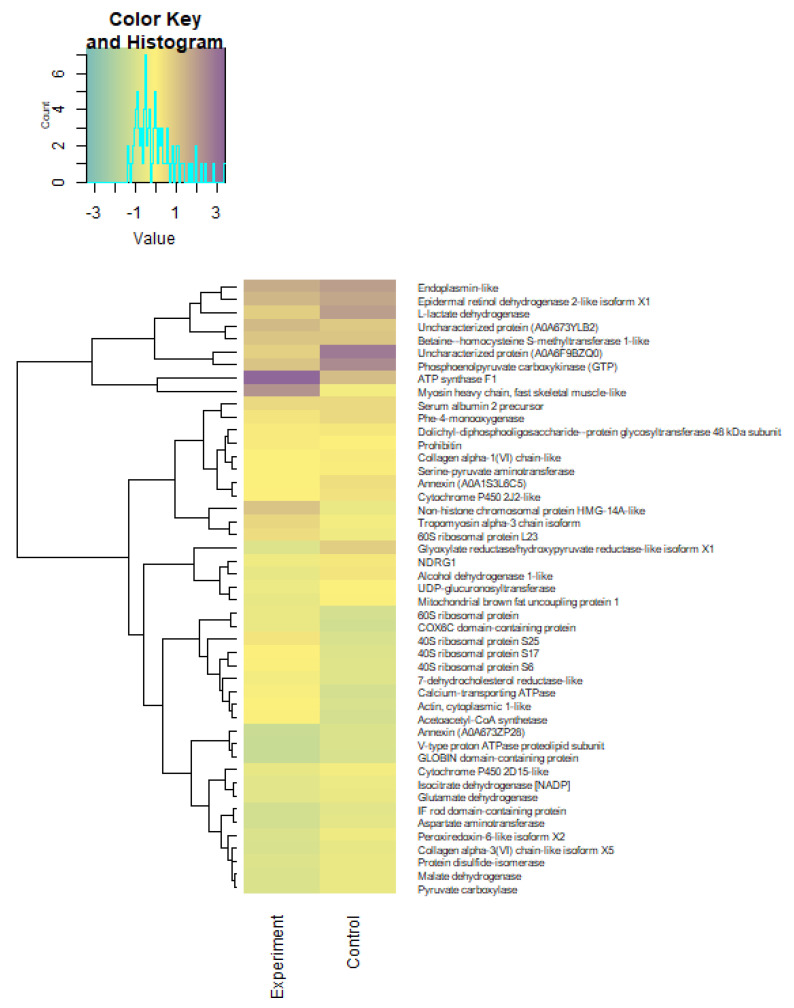
Heatmap LFQ Z-scores of normalized data of a “low concentration” proteome (group 3) in underyearlings of Atlantic salmon (*Salmo salar* L.) for control and experimental groups.

Using the Z-scores of normalized data, seven proteins (mainly belonging to the group of histone proteins) were isolated from 2018 proteins, the content of which was maximal in the experimental and control groups (Table 7). Quantitative changes in the proteome of underyearlings are shown in Figure 13, Figure 14 and Figure 15. Seventy of the 2018 identified proteins underwent significant quantitative changes, and they were conditionally divided into three groups (high concentration, mean concentration, and low concentration) to exclude the noise of the data with large values. It was noted that all seven isolated proteins at the previous stage of data analysis (Table 7) changed their concentration when exposed to laser radiation. The results showed that laser radiation had a strong effect on the content of histone proteins.

**Figure 16:**
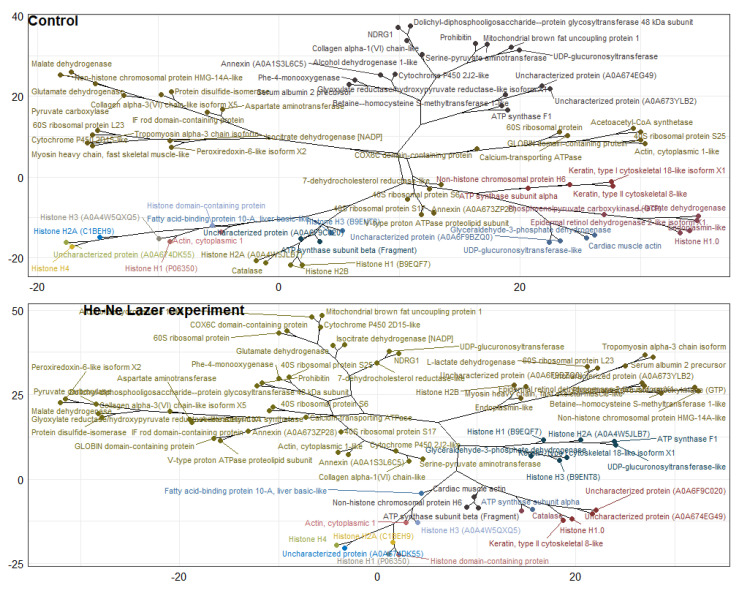
Hierarchical clustering visualizing as a “phylogenetic tree dendrogram” for control and experimental groups of underyearlings ofAtlantic salmon (*Salmo salar* L.). Note: 15 clusters are pick out.

A cluster analysis revealed significant changes in individual clusters upon exposure to laser radiation (Figure 16). The exception was the actin protein, cytoplasmic 1, the cluster of which did not change. It should be noted that the distances between individual clusters for high-concentration proteins (group 1) did not differ much under the influence of experimental radiation, while for mean and low concentrations (groups 2 and 3), there was a change in cluster n-th order.

The 70 proteins among the 2018 identified proteins underwent significant quantitative changes, and proteins were conditionally divided into three groups of “high concentration,” “mean concentration,” and “low concentration”. Cluster analysis determined significant changes in individual clusters upon exposure to He-Ne laser radiation (except for the actin protein cytoplasmic 1, the cluster of which did not change). The distances between individual clusters for high concentration proteins did not differ much under the influence of experimental radiation.

## 4. Discussion

Low mortality rates of Atlantic salmon eggs during embryonic development, as well as increased weight gain and lower morality rates of underyearlings compared to the control group, indicate the favorable effect of laser irradiation on the hatchery reproduction of salmon. These observations are consistent with the results of comparative biochemical studies of embryos and underyearlings, the development of which took place in the control and experimental basins.

A comparatively high total lipid content (19.78% dry weight) was found in mature eggs, with a predominance of reserve lipids as TAGs (9.56% dry weight; TAG/PL ratio: 4.31). It is known that readiness for fertilization, successful embryonic development, subsequent hatching of larvae, and viability before switching to exogenous nutrition depend on the starting levels of lipids and FAs in mature eggs of Atlantic salmon. Thus, we were dealing with high-quality mature eggs, with high developmental potential, taken from wild salmon.

Along with this, we found that the content of PLs (including the main fractions of PC and PEA and a minor fraction of LysoPC), as well as TAG and Chol in the laser-irradiated embryos at blastula stage were lower than in the control group. The changes in certain lipids indicated a compensatory reaction of the developing embryos aimed at ensuring the optimal activity of membrane-bound enzymes in response to laser irradiation. At the same time, the Chol/PL ratio and especially Chol esters/Chol (due to the decrease in Chol) increased in the experimental group at the blastula stage (up 1.16 and 1.56, respectively) compared with those in the control (1.10 and 1.33, respectively). Therefore, depending on the content of Chol, the permeability of the membrane to water and oxygen can change. The Chol/PL ratio is the main factor controlling the viscosity and fluidity of membranes. It is known that modification at the level of structural lipids (PLs, Chol) caused by a change in their content and proportion affects the viscosity of the lipid component of cell membranes. This ensures the optimal functioning of proteins and their functional activity, so the viability of the organism is maintained. The cleavage and blastula stages are the most sensitive stages of embryonic development of eggs, rapidly reacting and actively modifying the lipid systems of the body, especially under laser irradiation and its prolonged action.

In underyearling salmon that grew from embryos irradiated at the blastula stage, the physiological effects included a decrease in the content of storage lipids (TAG, Chol esters, and waxes) as well as in the indices of TAG/PL and Chol esters/Chol. There was a constant content of structural PLs, including their individual classes. At the same time, an increase in the value of the structural lipids to energetic lipids ratio as PL+Chol/TA +Chol esters+waxes ratio was established (from 0.28 in the control group to 0.33 in the experimental group). These changes indicate a higher metabolic rate in this group of juvenile salmon associated with the expenditure of energy lipids while maintaining (or slightly reducing) structural lipids. It is known that the rate of renewal (changes) in the content of structural PLs depends on the rate of DNA synthesis in the cell [36]. The regulation of the vital functions of underyearlings under prolonged laser irradiation is provided by metabolic rearrangements of the body’s lipid systems between storage and structural lipids. These are a consequence of changes in the ratios of individual lipid classes. At the same time, a decrease in the content of Chol and a lower Chol/PL ratio in experimental underyearlings can be considered one of the significant biochemical mechanisms of regulation of the physicochemical properties of biomembranes. The evidence suggests the value of an increase in the ionic permeability of biomembranes (for metabolites, water, and oxygen). An increase in the functional activity of membrane-bound enzyme systems and receptors, including those involved in the formation of a cellular immune response [37,38]. It should be noted that it is especially important to control the saturation of oxygen in water and its entry into the cells of the organism of juvenile fish in the factory rearing conditions.

It was found that among certain FAs, only the content of docosahexaenoic FA, 22:6(n-3), significantly decreased, and oleic FA, 18:1(n-9) increased in fish in the experimental group compared with the control group. It is interesting that the 20:5(n-3)/18:3(n-3) ratio was significantly higher and the 22:6(n-3)/20:5(n-3) ratio was lower in underyearlings in the experimental group. It is known that 22:6(n-3) FA is most sensitive to changes in the biochemical state of the internal environment of the body and external conditions. The arachidonic FA, 20:4(n-6), and 22:6(n-3) PUFAs are metabolic precursors of biologically active substances or lipid mediators, such as prostaglandins, thromboxanes, leukotrienes and protectins, D series resolvins, and maresin. These cause various physiological effects in the body, and they regulate many processes, such as muscle growth, immune responses, neurotransmitters, and hormonal functions.

At the blastula stage, differences were found in the level of activity of aerobic metabolism enzymes and some glucose oxidation pathways. The COX activity in the irradiated embryos was higher than in the control group. At the same time, the ratios of anaerobic and aerobic ATP synthesis (LDH/COX) indicated that the level of aerobic metabolism increased after laser treatment. According to previous studies, the mechanism of biostimulation by a low-energy laser can occur at the mitochondrial level [14,15,17]. In this case, mitochondria could be a special target of laser radiation, since they contain most of the cellular chromophores, while cytochrome c oxidase is probably the main mitochondrial photoacceptor [14,17,18]. It was found that COX activity increased after irradiation of yeast [16], cell lines [14], and purified enzyme [17]. Laser treatment of bovine sperm resulted in an overall increase in the affinity of COX to its substrate, as well as an increase in its activity, while COX activity and ATP content were positively correlated [18].

Since aerobic metabolism is more efficient than anaerobic metabolism, we can suggest an increase in energy supply in irradiated eggs and an intensification of metabolism, in particular the processes of biosynthesis. The high level of aerobic synthesis of ATP is of great importance in early ontogenesis when the intensive processes of biosynthesis take place [39]. Increased synthesis of cellular and mitochondrial proteins was established by irradiation of rat hepatocytes [40]. Under the influence of low-intensity laser radiation of loach embryos (*Misgurnus fossilis* L.), the number of mitochondria, lysosomes, and polysomes, and the channels of the granular endoplasmic reticulum increased, which reflected the activation of protein synthesis [9].

In addition, in our study, the eggs irradiated by the laser differed from the control eggs by a higher activity of G6PDH, a key enzyme of the pentose phosphate pathway (PPP), which indicates an intensification of this process [41]. In the course of the PPP, pentoses are formed, which are necessary to synthesize nucleic acids. Enhanced nucleic acid synthesis in cells after laser treatment was shown in human lymphocytes [42], rat hepatocytes [19], and isolated mitochondria [15].

In the course of PPP, reducing equivalents of NADPH also are formed, and they can be used in lipogenesis. In the irradiated eggs, the activity of 1GPDH also was higher than in the control group eggs. This might indicate an increase in the synthesis of glycerophosphate [43], which can be used in the synthesis of structural and reserve lipids.

There were no differences between the groups in the activity of the enzymes of glycolysis of aldolase and LDH, and their parameters were very low. Therefore, it can be assumed that the use of carbohydrates in the glycolysis of embryos did not differ, and in general, the intensity of this process was reduced. In a study on rainbow trout embryos at the stage of eye pigmentation, differences in the activity of anaerobic metabolism enzymes were found [20]. Thus, it can be assumed that, at the blastula stage, aerobic metabolism is most important. In a study of the embryogenesis of halibut *Scophthalmus maximus* L., it was found that the anaerobic pathway of ATP synthesis was dominant at the cleavage stage, and the aerobic pathway predominated in subsequent stages [44].

Since the use of carbohydrates in glycolysis was insignificant and did not differ between the groups, other energy substrates are probably used in the intensification of aerobic metabolism in experimental embryos and at this stage in general: fatty acids and amino acids. For example, it has been shown that in halibut embryogenesis, carbohydrates are mainly mobilized before the cleavage stage, and they are gradually replaced by amino acids from the cleavage stage to the gastrula stage and to fatty acids in the following days [44].

Newly obtained data on the proteome profile of fertilized eggs in the blastula stage of Atlantic salmon in the control and experimental groups revealed 131 proteins, of which 48 were significantly identified using MaxQuant and the UniProt database limited to species from the Salmonidae family. No qualitative differences in the proteomes of developing eggs in the blastula stage in the control and experimental groups were observed. The major protein was found to be phosvitin, the essential phosphoprotein of fish eggs and provisional molecule, which transferred from the maternal together with lipovitellin to maintain the growth and development of embryos [45,46]. Moreover, recent studies [46] of phosvitin revealed that it has an important role in the immunity of fish and participates in the protection of early embryos against pathogenic attacks. 

Differences were found in the proteomes in the blastula of Atlantic salmon in the control and experimental groups: phosvitin, cathepsin D-like, alpha-1-antitrypsin homolog, pentaxin, coagulation factor XIII B chain-like, serine protease-like protein, L-rhamnose-binding lectin CSL3-like, nucleoside diphosphate kinase, and complement C3, and the contents of which varied between the control and experimental groups. Thus, we assumed that the effects of He-–Ne laser irradiation on salmon eggs in the blastula stage affect metabolic processes (protein synthesis or innate immunity) involving these proteins. We draw attention to the observed differences in complement factor 3 (C3), which is present due to maternal transfer [47,48], and its multifunctional aspect in developing embryos. Primarily, C3 acts as an immune defense mechanism and has the alternative function of tagging apoptotic cells for removal [49,50], and it mediates stem cell commitment and differentiation, ossification, and signal transduction [51,52]. Moreover, in our early studies [53,54] on the dynamics of the activity of lysosomal proteinases in the embryogenesis of Atlantic salmon, a relatively high activity of cathepsin D (lysosomal endoproteinase) was detected in salmon eggs only 2 h after fertilization and in the blastula stage. The findings showed the hydrolysis of intracellular proteins stored in oogenesis, resulting in a pool of amino acids necessary for subsequent synthesis in the gastrulation stage.

In the present study, the qualitative and quantitative differences in the proteomes of underyearlings of Atlantic salmon from the control and experimental groups were established. The proteomes of underyearlings were represented by 2018 proteins, of which 49 were unique for the control group and 39 for the experimental group. Most of the proteins are involved in different molecular and physiological processes related to growth and development, including nerval and visual development, myogenesis, and immune system function. It was found that the most common proteoform in underyearlings was the ribosomal protein, which affects the activity of protein synthesis. Common protein families (in terms of frequency of occurrence) were mitochondrial proteins, dehydrogenases, synthases, cytochromes, myosin, and oxidases.

Among the 2018 identified proteins, only seven proteins (mainly belonging to the group of histone proteins) were isolated, the concentrations of which were abundant in the experimental and control groups. It was found that the effects of He–Ne laser radiation had a strong effect on the contents of histone proteins known to be responsible for gene expression patterns and in the epigenetic regulation of nucleosome mobility [55]. A recent study [56] showed that the chemical modifications of histones can influence gene transcription, as a certain sequence of that involves the change in metabolism and ion exchange with the environment. Furthermore, in [57] the molecular and physiological effects of exposure to different regimes of photoperiods as an external factor promoted full smolt development in juvenile salmon. In addition, histones are known as mediators of host defense [58], which is most important for newly hatched and developing underyearlings and thus essential for survival. It is known that a variety of core histone proteins have antimicrobial activity against a wide range of microorganisms, including Vibrio spp., the main pathogen of vibriosis in aquaculture [59]. It is necessary to point out that in certain cases, histone proteins and their changes may lead to inflammation and thrombosis [58]. 

## 5. Conclusions

Thus, irradiation with a He–Ne laser of the fertilized fish eggs of wild Atlantic salmon in the early fission stage promotes adaptive capacity and growth rate. The most prominent and favorable results are low mortality rates during embryonic development and increased weight gain and lower mortality rates for underyearlings developed from eggs irradiated by a He–Ne laser. The loss of fish products during artificial reproduction is one of the main indicators in the practice of fish farming. This is confirmed by changes in a number of interrelated indicators of lipid metabolism. The embryos in the blastula stage (experimental group) differed from the controls in terms of higher aerobic capacity and an increase in pentose phosphate pathway activity, likely indicating an intensification of biosynthesis. The absence of differences in metabolic enzyme activity in the muscles of the underyearlings suggests that there is no decline in energy metabolism due to irradiation.

Newly obtained data on the proteome profiles of fertilized eggs of Atlantic salmon in the blastula stage (control and experimental groups) revealed 131 proteins, of which 48 were significantly identified. The major protein was found to be phosvitin, the essential provisional phosphoprotein of fish eggs, which plays an important role in the immunity of fish and participates in the protection of early embryos against pathogenic attacks. Differences were found in the proteomes in the blastula of Atlantic salmon in the control and experimental groups. We draw attention to the observed differences in complement factor 3 (C3) and its multifunctional role in developing embryos, primarily in immune response. The proteomes of underyearlings were represented by 2018 proteins, of which 49 were unique for the control group and 39 for the experimental group. Most of the proteins are involved in different molecular and physiological processes related to growth and development, including nerve and visual development, myogenesis, and immune system function. The most common proteoform in underyearlings was the ribosomal protein, which affects the activity of protein synthesis. 

The results of this work obtained for Atlantic salmon cultured at the Vygskiy fish hatchery (White Sea region; 64.44° N, 34.54° E) are pioneering. Currently, we are repeating the experiment, and we are conducting a similar experiment using the same material but in the southern region of the Republic of Karelia (Lake Ladoga; 61.51° N, 30.26° E) in order to compare the effects of laser irradiation in the early embryonic development of Atlantic salmon on the subsequent growth and development of eggs and larvae in the northern and southern regions, considering that the timing of development, differentiation, and accumulation of mass during development can vary significantly depending on temperature and photoperiod.

## Figures and Tables

**Table 1:** Weight and length (M ± SE) characteristics of specimens taken for enzymes activity analysis.

Group	*n*	Weight, g	Fork Length, cm
Mature eggs			
	25	0.090 ± 0.001	-
Blastula stage			
Control	15	0.101 ± 0.007	-
LE	15	0.108 ± 0.003	-
Underyearlings			
Control	10	6.141 ± 0.323	8.140 ± 0.114
LE	10	7.394 ± 0.292	8.792 ± 0.133

**Table 2:** Certain metabolic ratios in mature (unfertilized) eggs, in blastula stage and in underyearlings of Atlantic salmon in control and experiment.

Stage	Mature Eggs	Blastula Stage (Control)	Blastula Stage (Experiment)	Underyearlings (Control)	Underyearlings (Experiment)
(n-3)/(n-6)	13.01 ± 0.16	11.99 ± 0.16	11.53 ± 0.14	3.06 ± 0.01	3.06 ± 0.02
16:0/18:1(n-9)	0.78 ± 0.02	0.68 ± 0.02	0.66 ± 0.02	1.07 ± 0.01	1.04 ± 0.01
18:1(n-9)/18:1(n-7)	5.18 ± 0.04	5.07 ± 0.22	5.39 ± 0.18	5.31 ± 0.08	5.32 ± 0.07
18:1(n-9)/18:0	2.75 ± 0.01	2.89 ± 0.1	2.98 ± 0.1	4.19 ± 0.03	4.15 ± 0.03
18:3(n-3)/18:2(n-6)	0.65 ± 0.01	0.56 ± 0.01	0.57 ± 0.01	0.24 ± 0	0.24 ± 0
20:4(n-6)/18:2(n-6)	0.71 ± 0.01	0.64 ± 0.03	0.6 ± 0.02	0.13 ± 0	0.12 ± 0
20:5(n-3)/18:3(n-3)	12.57 ± 0.07	11.32 ± 0.74	10.2 ± 0.5	3.58 ± 0.06	3.78 ± 0.1 *
22:6(n-3)/20:5(n-3)	1.89 ± 0.01	2.22 ± 0.09	2.29 ± 0.07	2.84 ± 0.06	2.62 ± 0.08 *

Note: *—significant differences between control and LE group (*p* < 0.05).

**Table 3:** The activity of energetic and carbohydrate enzymes (nmol × min^−1^ × mg^−1^ protein) in mature eggs, at the blastula stage and in underyearlings of Atlantic salmon in control and He-Ne lase experiment.

Parameter	Group	
Mature eggs		
LDH	3.78 ± 0.23 1.24 ± 0.09	
Aldolase		
Blastula stage		
	Control	Experiment
COX	65.21 ± 7.07	92.11 ± 9.43 *
LDH	29.41 ± 1.64	28.79 ± 2.71
G6PDH	14.32 ± 0.91	18.69 ± 2.04 *
GPDH	11.61 ± 1.57	18.04 ± 1.73 *
Aldolase	0.94 ± 0.08	1.01 ± 0.08
LDH/COX	0.51 ± 0.06	0.26 ± 0.03 *
Underyearlings		
	Control	Experiment
COX	22.16 ± 2.28	22.10 ± 4.01
LDH	5829.48 ± 364.83	5349.20 ± 393.27
Aldolase	173.20 ± 14.32	165.12 ± 8.25

Note: *—significant differences between control and LE group (*p* < 0.05). LDH—lactate dehydrogenase, COX—cytochrome c oxidase, G6PDH—glucose-6-phosphate dehydrogenase, GPDH—glycero-3-phosphate dehydrogenase.

**Table 4:** List of selected / most significant proteins.

Protein	Code	LOC	Organism	GO Molecular Function	GO Biological Process
Phosvitin	A0A1S3P927	LOC100136426	*Salmo salar*	Lipid transporter activity Nutrient reservoir activity	
Cathepsin D-like	A0A1S3QT99	LOC106596118	*Salmo salar*	Aspartic-type endopeptidase activity	
Alpha-1-antitrypsin homolog	A0A1S3SAE4	LOC106608112	*Salmo salar*	Extracellular space Cellular component	
Pentaxin	A0A1S3SCW1	LOC106608633	*Salmo salar*	Metal ion binding	
Coagulation factor XIII B chain-like	A0A1S3QWZ2	LOC106598077	*Salmo salar*	Lacks conserved residue(s) required for the propagation of feature annotation	
Serine protease-like protein	M4V2E4	LOC101448046	*Salmo salar*	Serine-type endopeptidase activity	Complement activation Notch signaling pathway
L-rhamnose-binding lectin CSL3-like	A0A1S3QXD6	LOC106597995	*Salmo salar*	Carbohydrate binding	
Nucleoside diphosphate kinase	B5XCJ8	NDKA	*Salmo salar*	ATP binding Nucleoside diphosphate activity	CTP biosynthetic process GTP biosynthetic process UTP biosynthetic process
Complement C3	A0A1S3QRL5	LOC106595495	*Salmo salar*	Endopeptidase inhibitor activity	Complement activation

**Table 5:** The importance of classifiers according to the “random forest” algorithm of the machine learning.

Protein	Mean Decrease Gini
Phosvitin	0.1781333
Cathepsin D-like	0.3953333
Alpha-1-antitrypsin homolog	0.1033333
Pentaxin	0.4060000
Coagulation factor XIII B chain-like	0.1740000
Serine protease-like protein	0.3880000
L-rhamnose-binding lectin CSL3-like	0.4153333
Nucleoside diphosphate kinase	0.3678667
Complement C3	0.1533333

Note: The Mean Decrease Gini represented the mean of the decrease in the Gini heterogeneity score, indicating the importance of each variable.

**Table 6:** List of unique proteins for the control and experimental group.

Protein	Code	LOC	Species	GO Molecular Function	GO Biological Process
***Control***					
40S ribosomal protein S26	B5XBS3	RS26	*Salmo salar*	Structural constituent of ribosome	Translation
Extended synaptotagmin-2-B-like isoform X2	A0A674ALB0	LOC106599471	*Salmo salar*	Lipid binding	Endoplasmic reticulum-plasma membrane tethering lipid transport
Membrane-spanning 4-domains subfamily A member 4A	B5XDT3	M4A4A	*Salmo salar*	Membrane	
Dolichyl-diphosphooligosaccharide--protein glycosyltransferase subunit KCP2	B5X788	KCP2	*Salmo salar*	Membrane	
Tubulin beta chain	A0A1S3PR98	LOC106586934	*Salmo salar*	GTPase activity GTP binding structural component of cytoskeleton	Microtubule-based process
Gap junction delta-4 protein-like	A0A1S3LVP6	LOC106568834	*Salmo salar*		Cell communication
Cellular nucleic acid-binding protein	B9EQ90	CNBP	*Salmo salar*	Nucleic acid binding Zinc ion binding	
Collagen alpha-1(XIV) chain-like	A0A1S3MK17	LOC106573054	*Salmo salar*	Collagen trimer	
Aldehyde dehydrogenase family 16 member A1	A0A1S3R7N3	aldh16a1	*Salmo salar*	Oxidoreductase activity, acting on the aldehyde or oxo group of donors, NAD or NADP as acceptor	
EH domain containing protein 1-like	A0A1S3SVN9	LOC106612088	*Salmo salar*	ATP binding Calcium ion binding GTP binding	
Protein-glutamine gamma-glutamyltransferase 2-like	A0A1S3L9D5	LOC106565224	*Salmo salar*	Metal ion binding Protein-glutamine gamma-glutamyltransferase activity	Peptide cross-linking
Glycylpeptide N-tetradecanoyltransferase	A0A1S3R7W7	LOC100380640	*Salmo salar*	glycylpeptide N-tetradecanoyltransferase activity	N-terminal protein myristoylation
Carnitine O-acetyltransferase-like	A0A1S3M6L2	LOC106570862	*Salmo salar*	transferase activity, transferring acyl groups	
Dipeptidyl peptidase 3	B5X435	DPP3	*Salmo salar*	dipeptidyl-peptidase activity metal ion binding metalloaminopeptidae activity	
DNA J homolog subfamily A member 2-like	A0A1S3KQ60	LOC106561246	*Salmo salar*	ATP binding heat shock protein binding metal ion binding unfolded protein binding	Protein folding response to heat
Heat shock 70 kDa protein 4-like isoform X4	A0A1S3RR20	LOC106604228	*Salmo salar*	ATP binding	
Heat shock 70 kDa protein-like	A0A1S3R3M8	LOC106599929	*Salmo salar*	ATP binding	
H/ACA ribonucleoprotein complex subunit	B5XA24	NOLA1	*Salmo salar*	RNA binding	Pseudouridine synthesis rRNA processing
Extracellular matrix protein 1-like	A0A1S3MVW9	LOC106575366	*Salmo salar*		signal transduction
Tropomyosin-1 alpha chain	B5X4E8	TPM1	*Salmo salar*	Actin binding	
Proactivator polypeptide	B5X4D6	SAP	*Salmo salar*		Adenylate cyclase-inhibiting G protein-coupled receptor signaling pathway regulation of lipid metabolic process sphingolipid metabolic process
Complement C2-like	A0A1S3NRD5	LOC106580826	*Salmo salar*	Serine-type endopeptidase activity	complement activation innate immune response
Signal recognition particle receptor subunit alpha-like isoform X1	A0A1S3NSP9	LOC106581092	*Salmo salar*	GTPase activity GTP binding signal recognition particle binding	SRP-dependent cotranslational protein targeting to membrane
Collagen alpha-1(I) chain	A0A1S3S6G4	LOC100286406	*Salmo salar*	Extracellular matrix structural Constituent metal ion binding	
Anoctamin	A0A1S3PDW1	ano5	*Salmo salar*	protein dimerization activity	
N-acetyltransferase 8-like	A0A1S3P7U0	LOC106583684	*Salmo salar*	N-acetyltransferase activity	
Spectrin beta chain	A0A1S3RFA5	LOC106602627	*Salmo salar*	Actin binding phospholipid binding structural constituent of cytoskeleton	Actin filament capping
CDGSH iron-sulfur domain-containing protein 2A	A0A1S3RH14	LOC106602754	*Salmo salar*	2 iron, 2 sulfurcluster binding Metal ion binding	Autophagy
Calpain-5-like isoform X2	A0A1S3T047	LOC106612883	*Salmo salar*	calcium-dependent cysteine-type endopeptidase activity	
cGMP-dependent protein kinase	A0A1S3N3S1	LOC106577002	*Salmo salar*	ATP binding cGMP binding cGMP-dependent protein kinase activity	
Cytochrome P450 3A27-like	A0A1S3QK62	LOC106593341	*Salmo salar*	Heme binding Iron ion binding Monooxygenase activity oxidoreductase activity, acting on paired donors, with incorporation or reduction of molecular oxygen, reduced flavin or flavoprotein as one donor, and incorporation of one atom of oxygen	
Si:ch211-152c8.4	A0A673XRM9	LOC100136426	*Salmo trutta*		
ER lumen protein-retaining receptor	A0A1S3Q507	LOC106589518	*Salmo trutta*	ER retention sequence binding	Protein retention in ER lumen protein transport
Uncharacterized protein	A0A674BI77	LOC115176156	*Salmo trutta*	Actin binding Calcium ion binding Microtubule binding	Intermediate filament cytoskeleton organization
HATPase_c domain-containing protein	A0A674BXP0	LOC115174523	*Salmo trutta*	ATP binding Unfolded protein binding	Protein folding
Dimethylaniline monooxygenase [N-oxide-forming]	A0A673YKU9	LOC115192436	*Salmo trutta*	Flavin adenine dinucleotide binding N,N-dimethylaniline monooxygenase activity NADP binding	
Uncharacterized protein	A0A674DSP5	tomm70	*Salmo trutta*	Transmembrane	
Transforming growth factor-beta-induced protein ig-h3	A0A673VXG8	LOC115167117	*Salmo trutta*	collagen binding extracellular matrix binding Integrin binding	Cell adhesion Cell population proliferation
Uncharacterized protein	A0A674DP36	LOC115198253	*Salmo trutta*	Zinc ion binding	Endoplasmic reticulum to Golgi vesicle-mediated transport intracellular protein transport
Ras-associating domain-containing protein	A0A673W873	LOC115197363	*Salmo trutta*	Adherens junction Maintenance signal transduction	
RNA helicase	A0A674AWY3	DDX5	*Salmo trutta*	ATP binding Nucleic acid binding RNA helicase activity	
Methionine synthase	A0A674BZ74	LOC115190130	*Salmo trutta*	Cobalamin binding Methionine synthase activity Zinc ion binding	Methylation pteridine-containing compound metabolic process
Uncharacterized protein	A0A6F9C1R4	CSTEINMANNI _LOCUS3550401	*Coregonus sp.*	Lipid transporter activity	
Uncharacterized protein (fragment)	A0A6F9AAX4	CSTEINMANNI _LOCUS1015781	*Coregonus sp. ‘balchen’*	Phosphatidylinositol binding	
Protein kinase domain-containing protein	A0A060Y1F8	GSONMT000	*Oncorhynchus mykiss*	ATP binding	
Myosin heavy chain	Q8JIP5	N/A	*Oncorhynchus keta*	Actin filament binding ATP binding motor activity	
Fibrillar collagen NC1 domain-containing protein	A0A4W5M2R8	N/A	*Hucho hucho (huchen)*	Extracellular matrix structural constituent	
Septin-type G domain-containing protein	A0A4W5N0E8	N/A	*Hucho hucho (huchen)*	GTP binding	
Uncharacterized protein	A0A4W5PNZ1	N/A	*Hucho hucho (huchen)*	Actin binding	
***He–Ne Laser experiment***					
Fructose-bisphosphatealdolase	O73866	ALDOB	*Salmo salar*	Fructose-bisphosphate aldolase activity	Glycolytic process
Enhancer of rudimentary homolog	B5X283	ERH	*Salmo salar*		Cell cycle
Outerdense fiber protein 2-like isoform X2	A0A1S3R511	LOC106600419	*Salmo salar*		Negative regulation of cilium assembly
Lumican	A0A1S3N0X4	LOC106576454	*Salmo salar*		Collagen fibril organization visual perception
Cathepsin M	Q70SU8	salarin	*Salmo salar*	Cysteine-type endopeptidase inhibitor activity protease binding	
Uncharacterized protein	A0A1S3QJQ6	LOC106593464	*Salmo salar*	Catalytic activity	
Tetratricopeptide repeat protein 36	A0A1S3NVC8	ttc36	*Salmo salar*		
Erythrocyte band 7 integral membrane protein-like	A0A1S3KZT9	LOC106563232	*Salmo salar*		
SURF1-like protein	B5XGV8	SURF1	*Salmo salar*	Cytochrome-c oxidase activity	Embryonic organ development spinal cord motor neuron differentiation
Tropomyosin alpha-3 chain-like isoform X3	A0A1S3NNW5	LOC106580396	*Salmo salar*	Actin binding	
Syntenin-1	B5X137	SDCB1	*Salmo salar*		
Serine palmitoyl transferase 1 isoform X1	A0A1S3MCX2	sptlc1	*Salmo salar*	Pyridoxal phosphate binding Transferase activity	Biosynthetic process
Cytochrome P450 2F3-like	A0A1S3QRX0	LOC106595913	*Salmo salar*	heme binding iron ion binding oxidoreductase activity, acting on paired donors, with incorporation or reduction of molecular oxygen, reduced flavin or flavoprotein as one donor, and incorporation of one atom of oxygen	
SWI/SNF-related matrix-associated actin-dependent regulator of chromatin subfamily E member 1-like isoform X1	A0A1S3RAD2	LOC106601263	*Salmo salar*	DNA binding	ATP-dependent chromatin remodeling
Myosin heavy chain, fast skeletal muscle-like	A0A1S3QIX9	LOC106593179	*Salmo salar*	Actin filament binding ATP binding motor activity	
Probable 2-oxoglutarate dehydrogenase E1 component DHKTD1, mitochondrial	A0A1S3MIZ7	LOC106573060	*Salmo salar*	oxoglutarate dehydrogenase (succinyl-transferring) activity thiamine pyrophosphate binding	Tricarboxylic acid cycle
Torsin-1A-interacting protein 2-like	A0A1S3KK08	LOC106560502	*Salmo salar*		
Alkaline phosphatase	A0A1S3LDJ3	LOC106565890	*Salmo salar*	Alkaline phosphatase activity	
LIM and SH3 domain protein 1	A0A1S3S575	LOC106607249	*Salmo salar*	metal ion binding	Ion transport
Peptidase M20 domain-containing protein 2	A0A1S3S7W2	pm20d2	*Salmo salar*	Hydrolase activity	
Putative all-trans-retinol 13,14-reductase	A0A1S3S561	LOC100196171	*Salmo salar*		
Myosin-10-like isoform X12	A0A1S3L623	LOC106564681	*Salmo salar*	Actin filament binding ATP binding motor activity	
Annexin	B5XAE0	ANXA5	*Salmo salar*	Calcium-dependent phospholipid binding calcium ion binding	Negative regulation of coagulation
Catenin delta-1-like isoform X7	A0A1S3RRP7	LOC106604654	*Salmo salar*	Cadherin binding	Cell–cell adhesion
Granulins-like isoform X2	A0A1S3RAE7	LOC106601556	*Salmo salar*		
Utrophin-like isoform X7	A0A1S3S9Q6	LOC106608002	*Salmo salar*	Actin binding Zinc ion binding	
Apolipoprotein B-100-like	A0A1S3M854	LOC106571178	*Salmo salar*	Lipid transporter activity	
RNA-binding protein 4B	B5DG79	LOC106577685	*Salmo salar*	RNA binding	
Keratin, type I cytoskeletal 13-like isoform X2	A0A1S3L7P6	LOC106564960	*Salmo salar*	Structural molecule activity	
Fibrillar collagen NC1 domain-containing protein	A0A673ZWM6	N/A	*Salmo trutta*	Extracellular matrix structural constituent	
GRIP domain-containing protein	A0A674ABA3	LOC115199935	*Salmo trutta*		
Vacuolar proton pump subunit B	A0A673WHM8	LOC115193839	*Salmo trutta*	ATP binding	ATP metabolic process
Uncharacterized protein	A0A674BVJ8	LOC115166471	*Salmo trutta*	Calcium ion binding	Endocytosis
Uncharacterized protein	A0A674DB91	TPM1	*Salmo trutta*	actin binding	
SERPIN domain-containing protein	A0A060XBJ0	GSONMT00058278001	*Oncorhynchus mykiss*	Serine-type endopeptidase inhibitor activity	Negative regulation of complement activation regulation of blood coagulation
Uncharacterized protein	A0A4W5LNI8	N/A	*Hucho hucho*		
Coagulation factor X	A0A4W5JVR3	F10	*Hucho hucho*	Calcium ion binding Serine-type endopeptidase activity	Blood coagulation
Uncharacterized protein	A0A4W5MR86	N/A	*Hucho hucho*		
NADH-ubiquinone oxidoreductase 75 kDa subunit, mitochondrial	A0A4W5K6Y5	N/A	*Hucho hucho*	4 iron, 4 sulfur cluster binding electron transfer activity NADH dehydrogenase	ATP synthesis coupled electron transport

**Table 7:** The content of highly abundant proteins in underyearlings of Atlantic salmon (*Salmo salar* L.).

Protein	Code	Species	GO Molecular Function	GO Biological Process	Control (iBAQ)	He–Ne Laser Experiment (iBAQ)
Uncharacterized protein	A0A674DK55	*Salmo turtta*	DNA binding protein heterodimerization activity		19647000000	16077000000
Histone H4	A0A674C0Y3	*Salmo trutta*	DNA binding protein heterodimerization activity	DNA-templated transcription, initiation	19301000000	12952000000
Histone H2A	C1BEH9	*Oncorhynchus mykiss*	DNA binding protein heterodimerization activity	Defense response to Gram-positive bacterium Innate immune response Membrane disruption in other organisms	14375000000	7603900000
Histone H1	P06350	*Oncorhynchus mykiss*	DNA binding	Defense response to bacterium Nucleosome assembly	8659100000	5947400000
Histone H3	A0A4W5QXQ5	*Hucho hucho*	DNA binding protein heterodimerization activity		8337600000	2860100000
Histone domain-containing protein	A0A060WQS0	*Oncorhynchus mykiss*	DNA binding protein heterodimerization activity		3103700000	5338700000
Actin, cytoplasmic 1	O42161	*Salmo salar*	ATP binding		2906100000	3423800000

## Data Availability

All data are presented in the paper.
